# Dynamic model updating (DMU) approach for statistical learning model building with missing data

**DOI:** 10.1186/s12859-021-04138-z

**Published:** 2021-04-29

**Authors:** Rahi Jain, Wei Xu

**Affiliations:** 1 Biostatistics Department, Princess Margaret Cancer Research Centre, Toronto, ON Canada; 2 Dalla Lana School of Public Health, University of Toronto, Toronto, ON Canada

**Keywords:** Missing data, Bayesian regression, Hierarchical clustering, Model updating, Dynamic model updating

## Abstract

**Background:**

Developing statistical and machine learning methods on studies with missing information is a ubiquitous challenge in real-world biological research. The strategy in literature relies on either removing the samples with missing values like complete case analysis (CCA) or imputing the information in the samples with missing values like predictive mean matching (PMM) such as MICE. Some limitations of these strategies are information loss and closeness of the imputed values with the missing values. Further, in scenarios with piecemeal medical data, these strategies have to wait to complete the data collection process to provide a complete dataset for statistical models.

**Method and results:**

This study proposes a dynamic model updating (DMU) approach, a different strategy to develop statistical models with missing data. DMU uses only the information available in the dataset to prepare the statistical models. DMU segments the original dataset into small complete datasets. The study uses hierarchical clustering to segment the original dataset into small complete datasets followed by Bayesian regression on each of the small complete datasets. Predictor estimates are updated using the posterior estimates from each dataset. The performance of DMU is evaluated by using both simulated data and real studies and show better results or at par with other approaches like CCA and PMM.

**Conclusion:**

DMU approach provides an alternative to the existing approaches of information elimination and imputation in processing the datasets with missing values. While the study applied the approach for continuous cross-sectional data, the approach can be applied to longitudinal, categorical and time-to-event biological data.

**Supplementary Information:**

The online version contains supplementary material available at 10.1186/s12859-021-04138-z.

## Background

Missing data is a ubiquitous problem across non-experimental, field-based studies such as genomic, epidemiological, and social science studies. Conventionally, complete case analysis (CCA) and imputation are two approaches to address the missingness. CCA uses samples with complete data for model building, which limits its application to scenarios with large samples of complete data. Further, CCA provides biased estimates in cases when data is not missing completely at random (MCAR) [1].

Imputation approach can handle samples without complete data by replacing missing data with either single values (Single imputation) or multiple values (Multiple imputation). Single imputation technique adds single plausible value to each missing value and creates a single imputed dataset. Single imputation approach treats imputed values as an actual value rather than an estimate with standard error value during the downstream analysis, which creates a potential bias in the results [2]. Mean imputation is one of the most straightforward imputation techniques. It replaces the missing values of a predictor with the mean value of the observed data of the predictor. The main disadvantages are that it underestimates the variance of the predictor and ignores the relationship between the predictors [3, 4]. Regression imputation is another technique in which the predictor with missing value is regressed on the predictors with non-missing values. Finally, the missing values of the predictor are estimated from the regression model [5]. However, the technique relies on the linear relationship [6], which may affect the model quality.

Multiple imputation approach provides more unbiased estimates as compared to single imputation approach as it considers the uncertainty in estimates. Multiple imputation approach assigns multiple plausible values to every missing value, which creates multiple imputed datasets. Each dataset undergoes analysis and results are pooled using Rubin’s rules [7]. The *MICE* package in R is one of the popular packages for performing multiple imputations [8]. It provides many multiple imputation approaches like predictive mean matching, Bayesian regression, and linear regression. However, the multiple imputation approach still cannot provide unbiased estimates for all scenarios [9].

In non-linear relationships among the predictors, various machine learning-based techniques are used to perform imputation. K-Nearest Neighbors (K-NN) is one of the machine learning technique used for imputation. For any predictor with missing values, K-NN tries to identify the *k* nearest neighbors for each missing value using the predictors with non-missing data. The missing value is imputed using the values of the *k* nearest neighbors [10]. K-means clustering segregates the complete dataset (including missing values) into *k* clusters. Then, K-NN algorithm is applied in each cluster to impute the missing values in the cluster [11]. However, in many cases, K-NN and K-means based approaches could perform poorly as compared to other approaches [12, 13]. MissForest technique uses random forest for imputing the missing data to overcome the limitations of regression-based imputation methods [14].

In many real-world scenarios, data collection is not simultaneous. Instead, it happens over time. CCA and imputation-based approaches have to wait for the completion of the data collection process. In scenarios of high throughput data, data storage can be an issue [15]. This paper successfully proposes an alternative, i.e., dynamic model updating (DMU) approach of analyzing the dataset with missing values. DMU analyses multiple smaller datasets obtained from the original dataset rather than the original dataset and allowing estimate updating with every single analysis. The paper is organized as follows. ‘Methodology’ section describes the DMU algorithm; the model performance is evaluated and demonstrated using simulation and real dataset studies in the “Simulation Studies” and “Real Data Study” sections, respectively. Finally, the ‘Conclusion and Discussion’ section concludes the paper and discusses the limitations of the study.

## Results

The performance of DMU is evaluated and compared with CCA and PMM approach for both the simulated datasets and real data studies.

### Simulation studies

We use simulation studies to evaluate model performance. In the simulation studies, data is generated from the following regression model:

1 $$\begin{array}{*{20}c} {y = \beta_{0} + \beta_{1} x_{1} + \cdots + \beta_{p} x_{p} + \epsilon } \\ \end{array}$$

where *ε* ~ N(0, 0.25) is noise in the model and x_1_,…,x_p_ are predictors. The values for predictors x_1_ and x_2_ is drawn from beta (~ Beta(7, 2)) and uniform (~ U(0, 2)) distribution respectively, while the values for predictors x_3_,…,x_p_ is drawn from normal distribution(~ N(0, 1)). Coefficient values for x_1_, x_2_ and x_3_ are 0.2, 0.3 and 0.4 respectively. The remaining predictors have zero coefficient values. The correlation matrix is designed to add multicollinearity to the model. The predictors {x_1_,…,x_5_} are randomly assigned correlation values between [− 0.5, 0.5] with replacement and zero correlation value is assigned to all other cases as shown below.

$$\left[ {\begin{array}{*{20}c} {\begin{array}{*{20}c} {x_{1} x_{1} } & {x_{1} x_{2} } & . \\ {x_{2} x_{1} } & {x_{2} x_{2} } & . \\ . & . & . \\ \end{array} } & {\begin{array}{*{20}c} . & {x_{1} x_{5} } & {\begin{array}{*{20}c} . & . \\ \end{array} } \\ . & {x_{2} x_{5} } & {\begin{array}{*{20}c} . & . \\ \end{array} } \\ . & . & {\begin{array}{*{20}c} . & . \\ \end{array} } \\ \end{array} } \\ {\begin{array}{*{20}c} . & . & . \\ {x_{5} x_{1} } & {x_{5} x_{1} } & . \\ {\begin{array}{*{20}c} {x_{6} x_{1} } \\ . \\ {x_{p} x_{1} } \\ \end{array} } & {\begin{array}{*{20}c} {x_{6} x_{1} } \\ . \\ . \\ \end{array} } & {\begin{array}{*{20}c} . \\ . \\ . \\ \end{array} } \\ \end{array} } & {\begin{array}{*{20}c} . & . & {\begin{array}{*{20}c} . & . \\ \end{array} } \\ . & {x_{5} x_{5} } & {\begin{array}{*{20}c} . & . \\ \end{array} } \\ {\begin{array}{*{20}c} . \\ . \\ . \\ \end{array} } & {\begin{array}{*{20}c} {x_{6} x_{5} } \\ . \\ . \\ \end{array} } & {\begin{array}{*{20}c} . & . \\ . & . \\ . & . \\ \end{array} } \\ \end{array} } \\ \end{array} } \right] = \left[ {\begin{array}{*{20}c} 1 & {\left[ { - 0.5,{ }0.5} \right]} & . & . & {\left[ { - 0.5,{ }0.5} \right]} & 0 & . \\ {\left[ { - 0.5,{ }0.5} \right]} & 1 & . & . & {\left[ { - 0.5,{ }0.5} \right]} & 0 & . \\ {\left[ { - 0.5,{ }0.5} \right]} & {\left[ { - 0.5,{ }0.5} \right]} & 1 & . & {\left[ { - 0.5,{ }0.5} \right]} & 0 & . \\ {\left[ { - 0.5,{ }0.5} \right]} & {\left[ { - 0.5,{ }0.5} \right]} & . & 1 & {\left[ { - 0.5,{ }0.5} \right]} & 0 & . \\ {\left[ { - 0.5,{ }0.5} \right]} & {\left[ { - 0.5,{ }0.5} \right]} & . & . & 1 & 0 & . \\ 0 & 0 & 0 & 0 & 0 & 1 & . \\ . & . & . & . & . & . & 1 \\ 0 & 0 & 0 & 0 & 0 & 0 & 0 \\ \end{array} } \right]$$

A multivariate normal distribution generates data for 20, 25, 30 and 100 predictors (p) in the simulated dataset, D. Two scenarios are created to test the performance of different methods. In the first scenario, the training dataset contains some complete rows (SCR). Training data comprises of 3150 samples with each predictor having 80% of its values MCAR and 50 samples of complete data across all predictors. Test data consisted of 1000 samples of complete data across all predictors. In the second setting, the training dataset has no complete row (NCR). Training data comprised of 3150 samples with each predictor having 80% of its values MCAR. Test data consisted of 1000 samples of complete data across all predictors.

DMU method is used to build the model and its performance is estimated. Prior distributions are defined as follows:

2 $$\begin{array}{*{20}c} {\epsilon \sim N\left( {0,\sigma^{2} } \right)} \\ \end{array}$$

3 $$\begin{array}{*{20}c} {\sigma^{ - 2} \sim Gamma\left( {\frac{5}{2},\frac{50}{2}} \right)} \\ \end{array}$$

4 $$\begin{array}{*{20}c} {\beta \sim N\left( {0,100} \right) | \beta \varepsilon \left\{ {\beta_{1} , \ldots ,\beta_{p} } \right\}} \\ \end{array}$$

Markov Chain Monte Carlo (MCMC) is used to generate the posterior distribution of parameters in the model using *MCMC* package in R [16]. Total 6000 iterations are performed and the first 1000 are used as burn-in iterations. A constraint is used to segment Dataset D such that *d*_*i*_ with sample size to predictor set ratio greater than or equal to two is used for model building. Because optimal *k* is not known, hence the genetic algorithm is used for selecting *k*. The performance of the DMU method is compared with simple linear regression (SLR), k-Nearest Neighbors based imputation (kNN), simple linear regression combined with imputation (SLRM) and random forest based imputation (RF) using simulated data. In the case of SLRM, the predictive mean matching (PMM) based imputation method provided by *the MICE package* of R [8] is used to impute missing data in the dataset. *VIM* package and *missForest* packages of R are used for kNN imputation and RF imputation, respectively [14, 17]. The performance of different methods is evaluated using mean square error (MSE) between the estimated outcome and the actual outcome in the test data. The reported performance is normalized with mean imputation MSE performance. The GA package in R [18] is used for the genetic algorithm.

Simulation datasets (S = 30) are created with the abovementioned settings and the overall performance of each method is measured. Table 1 and Additional file 1: Table S1 shows the performance results of the five methods. The study shows that the DMU has a lower or comparable MSE as compared to the MSE of SLR and SLRM. In SCR settings, SLRM gave the worst performance, and kNN gave the best performance, while DMU performance is either similar or better than the SLR and RF method. In NCR settings, RF gave the best performance, but DMU performance is better or similar to SLRM. Overall, these results suggest that the DMU can develop better or at par models as compared to SLR and SLRM based models for datasets with values missing completely at random. The results are validated in higher feature space (*p* = 100), where DMU MSE for SCR is 0.16 (1.27 MSE(DMU)/MSE(mean imputation)) but SLRM MSE for SCR is 0.23. KNN and RF imputation performed better with MSE of 0.13. In case of NCR, DMU MSE performance of 0.13 is comparable with RF imputation MSE performance of 0.13 and better than SLRM MSE performance of 0.47.

**Table 1:** MSE performance of different methods in simulated datasets after adjusting for mean imputation performance

Settings	p	Average (MSE (Method)/MSE (Mean Imputation) (S = 30)				
		SLR (95% CI)	KNN (95% CI)	SLRM (95% CI)	RF (95% CI)	DMU (95% CI)
SCR	20	1.03 (0.9–1.17)	1 (0.88–1.12)	2.03 (1.81–2.26)	1.17 (1.06–1.28)	0.99 (0.88–1.1)
	25	1.2 (1.05–1.35)	0.97 (0.86–1.08)	2.01 (1.8–2.22)	1.12 (1.02–1.22)	1.08 (0.97–1.2)
	30	1.44 (1.24–1.63)	0.98 (0.86–1.09)	1.95 (1.77–2.13)	1.1 (1.01–1.18)	0.98 (0.90–1.06)
NCR	20	–	–	1.89 (1.69–2.1)	1.1 (1.01–1.2)	1.29 (1.19–1.38)
	25	–	–	2.07 (1.85–2.29)	1.12 (1.03–1.22)	1.59 (1.47–1.71)
	30	–	–	1.83 (1.62–2.04)	1.05 (0.96–1.13)	1.73 (1.6–1.87)

*SLR* Simple Linear Regression, *KNN* k Nearest Neighbors based Imputation, *SLRM* Simple Linear Regression combined with imputation, *RF* Random Forest-based Imputation, *DMU* Dynamic Model Updating, *SCR* Some Complete Rows in training data, *NCR* No Complete Rows in training data, *CI* Confidence Interval

Table 2 provides the computation time for different methods on a system with processor Intel® Core(TM) i7-8750H CPU@2.20 GHz with 16 GB RAM on a Windows 10 64-bit operating system. We use the SCR scenario and three different feature spaces (*p*), i.e., 20, 25 and 30, to estimate time. It is found that DMU with optimized hyperparameters and SLRM takes a similar computation time which is less than random forest and kNN based imputation but more than SLR.

**Table 2:** Comparison of computation time for different methods in SCR scenario

p	Scenario	Computation time (s)				
		SLR	KNN	SLRM	RF	DMU
20	SCR	0.008	36.719	3.527	52.926	0.866
25	SCR	0.044	50.989	5.637	76.500	1.376
30	SCR	1.080	72.720	8.161	89.743	10.794

*SLR* Simple Linear Regression, *KNN* k Nearest Neighbors based Imputation, *SLRM* Simple Linear Regression combined with imputation, *RF* Random Forest-based Imputation, *DMU* Dynamic Model Updating, *SCR* Some Complete Rows in training data

### Real data studies

Furthermore, the study compares the proposed regression method with SLR and SLRM using two real-world datasets. Dataset I is Community Health Status Indicators dataset which contains USA county-level data on various demographics and health parameters to help in making informed decisions in combating obesity, heart disease and cancer [19]. The dataset contains data on 578 features for 3141 US counties. Dataset II is Study of Women’s Health Across the Nation, 2006–2008 dataset which contains multi-site data for middle-aged women in the USA on various *physical, biological, psychological and social* parameters [20]. The dataset contains data on 887 features for 2245 respondents.

These datasets are processed and cleaned to remove textual or categorical variables. One of the shortlisted variables is used as the outcome variable and remaining variables are used as predictors. Different scenarios are created using these two datasets, as shown in Table 3. The maximum correlation allowed between the predictors in each scenario is ± 0.52. Different predictors have a different percentage of missing values; thus, the maximum percentage of missing values is defined for each scenario. For example, in Scenario 1, predictors up to 10% of missing values is selected for model building. It is possible to have datasets in real-world settings where no single row has data for all the predictors. Hence, the study tried to recreate the settings by testing the performance of the methods in two different settings. In the first setting, some complete rows (SCR) are added into training dataset. In the second setting, no complete row (NCR) is added in the training dataset. In both settings, the test dataset only comprised of compete rows. Since SLR could be performed only on rows with complete data, so this method is not applied to scenarios with no complete rows in the training dataset. All three methods are compared based on their prediction performance in the test datasets.

**Table 3:** Summary of the real datasets

Scenario	Dataset	Correlation	Maximum missing (%)	Complete row	Predictors	Outcome variable	Sample size (n)		
							Total	Train	Test
1	I	± 0.52	10	Yes	27	Percentage of unhealthy days	2596	1432	1164
2		± 0.52	20	Yes	30	Percentage of unhealthy days	2596	1571	1025
3		± 0.52	30	Yes	32	Percentage of unhealthy days	2596	1793	803
4		± 0.52	10	No	27	Percentage of unhealthy days	2596	267	2329
5		± 0.52	20	No	30	Percentage of unhealthy days	2596	546	2050
6		± 0.52	30	No	32	Percentage of unhealthy days	2596	990	1606
7	II	± 0.52	10	Yes	5	Body Mass Index	1947	1000	947
8		± 0.52	20	Yes	20	Body Mass Index	1947	1162	785
9		± 0.52	30	Yes	21	Body Mass Index	1947	1242	705
10		± 0.52	10	No	5	Body Mass Index	1947	52	1895
11		± 0.52	20	No	20	Body Mass Index	1947	376	1571
12		± 0.52	30	No	21	Body Mass Index	1947	536	1411

Table 4 and Additional file 2: Table S2 provides the performance of different methods on two real datasets. The results are like those obtained in the simulated datasets. The proposed DMU approach provides better or at par MSE performance as compared to other methods. The performance is consistent across different proportions of missing data, but increased sample size in training data improves the performance of all the approaches. NCR seems to increase the MSE of the methods.

**Table 4:** Performance of different methods on the real datasets

Scenario	Dataset	MSE (Method)/MSE (Mean Imputation)				
		SLR	kNN	SLRM	RF	DMU
1	I	0.36	1.05	0.37	2.41	0.16
2	I	0.34	1.08	0.43	1.56	0.15
3	I	0.43	0.95	0.74	1.11	0.07
4	I	–	–	1.69	1.42	0.71
5	I	–	–	0.91	1.27	0.19
6	I	–	–	1.18	1.51	0.05
7	II	0.84	0.96	0.84	1.00	0.84
8	II	0.33	0.99	0.38	0.62	0.32
9	II	0.25	0.88	0.31	0.57	0.24
10	II	–	–	0.44	0.85	0.35
11	II	–	–	0.87	0.58	0.33
12	II	–	–	0.69	0.50	0.36

*SLR* Simple Linear Regression, *KNN* k Nearest Neighbors based Imputation, *SLRM* Simple Linear Regression combined with imputation, *RF* Random Forest-based Imputation, *DMU* Dynamic Model Updating

### Real data studies: genomic data

The study also compares the proposed regression method with SLR, SLRM, kNN and RF using a real-world genomic dataset. A Genomics of Drug Sensitivity in Cancer (GDSC) dataset containing copy number variations (CNV) in 24,503 genes and inhibitory concentrations (IC50) of cancer drugs for 946 cell line samples is used [21]. We selected Devimistat (CPI-613) drug IC50 as the clinical outcome and CNV as input feature space. The drug is known to reduce the aggressiveness of pancreatic cancer by inhibiting the tricarboxylic acid cycle and Is currently in Phase III clinical trial [22].

The dataset is processed and cleaned to remove input features with duplicated values, high correlation and no missing value. The reduced dataset contains 42 input features with 911 samples. The dataset is randomly split into 80% training data and 20% test data. Around 30% of the input feature values from each input features is randomly removed from the training data. The performance of the different method is compared for both SCR and NCR scenarios over five trials. In the case of SCR, 50 samples are randomly added to training data. It is found that DMU outperformed all other methods (Table 5).

**Table 5:** Performance of different methods on the real genomic dataset

Technique	Average (MSE (Method)/MSE (Mean Imputation) (S = 5)	
	NCR (95% CI)	SCR (95% CI)
SLR (95% CI)	–	7.24 (1.03–13.46)
KNN (95% CI)	–	1.00 (0.99–1.01)
SLRM (95% CI)	0.98 (0.97–1.00)	0.98 (0.97–1.00)
RF (95% CI)	1.03 (0.99–1.06)	1.01 (0.99–1.02)
DMU (95% CI)	0.92 (0.86–0.98)	0.97 (0.92–1.02)

*SLR* Simple Linear Regression, *KNN* k Nearest Neighbors based Imputation, *SLRM* Simple Linear Regression combined with imputation, *RF* Random Forest-based Imputation, *DMU* Dynamic Model Updating, *SCR* Some Complete Rows in training data, *NCR* No Complete Rows in training data, *CI* Confidence Interval

## Discussion

Handling missing data during model building is a challenge that this study addresses using a new perspective. DMU allows building the model from samples with partial information rather than removing samples with partial information or imputing information. DMU performance is better than complete case analysis and predictive mean matching based imputation when applied in linear regression.

The proposed method has certain limitations. First, the comprehensiveness of the DMU testing is limited. The model is not tested on different datasets like datasets containing categorical outcome, time to event outcome, categorical predictors. Similarly, it did not consider high correlation variables, interaction terms and different continuous distributions like exponential and logarithmic. Thus, our approach could be considered for datasets with continuous marginal features and outcome with low correlation among the features. Future studies have scope to determine the robustness of the DMU in different data settings.

Another limitation of the study is the computational intensiveness, especially in cases where the number of subgroups is not pre-defined. In such cases, computational resources need to be spent identifying the best value of k by creating multiple models. The study uses a genetic algorithm to address the problem. Various other optimization algorithms like swarm optimization and simulated annealing can be explored in addressing the problem.

## Conclusion

An innovative approach is proposed for building statistical models with missing data. DMU approach divides the dataset with missing values into smaller subsets of complete data followed by preparing and updating the Bayesian model from each of the smaller subsets. The approach provides a different perspective of building models with missing data using available data as compared to the existing perspective in the literature of either removing missing data or imputing missing data. The approach is more flexible as compared to existing approaches as it can update the old models with new data without a need to retain the old data. Secondly, DMU does not depend on the association among the predictors for imputing data. Hence, MU can update the models even when the new dataset contains an incomplete list of predictors.

## Methodology

In this section, first CCA and Predictive Mean Matching (PMM) based imputation are described, followed with the dynamic model updating (DMU) method.

### Complete case analysis (CCA)

Complete Case Analysis is a common approach used in handling the missing data. This approach omits all the samples with missing data. CCA builds a statistical model from the remaining samples with complete data (or, complete cases). The approach performance is affected when many samples are omitted [23], or data is not missing completely at random [1].

### Predictive mean matching (PMM) based imputation

Predictive Mean Matching is a common approach for imputing missing data in MCAR cases. It is a robust approach which assigns an observed value to the missing case. In this approach, the predictor with missing values (X_miss_) is regressed on the predictor/s with complete values (X_obs_):

5 $$\begin{array}{*{20}c} {X_{miss} = \beta_{0} + \beta_{1} X_{obs} + \ldots } \\ \end{array}$$

where β = β_o_, β_1_,… are estimates of regression coefficients and used to get estimated values of X_miss_. Once the estimated values of X_miss_ are obtained, these values are replaced with the closest observed value of X_miss_ in the dataset. Multiple imputed datasets are created by randomly sampling one of the *k* closest value, instead of the closest value, for each of estimated value of X_miss_ in the dataset. *k* is usually in the range of 1–10. This approach is implemented in *MICE* package in R, where the default value of *k* is 5 [8]. One of the limitations of this approach is that it always imputed data from the observed values. Thus, in cases where the missing values are in the tail of a distribution, PMM may have biased imputation [24].

### Dynamic model updating (DMU) approach

**Fig. 1:**
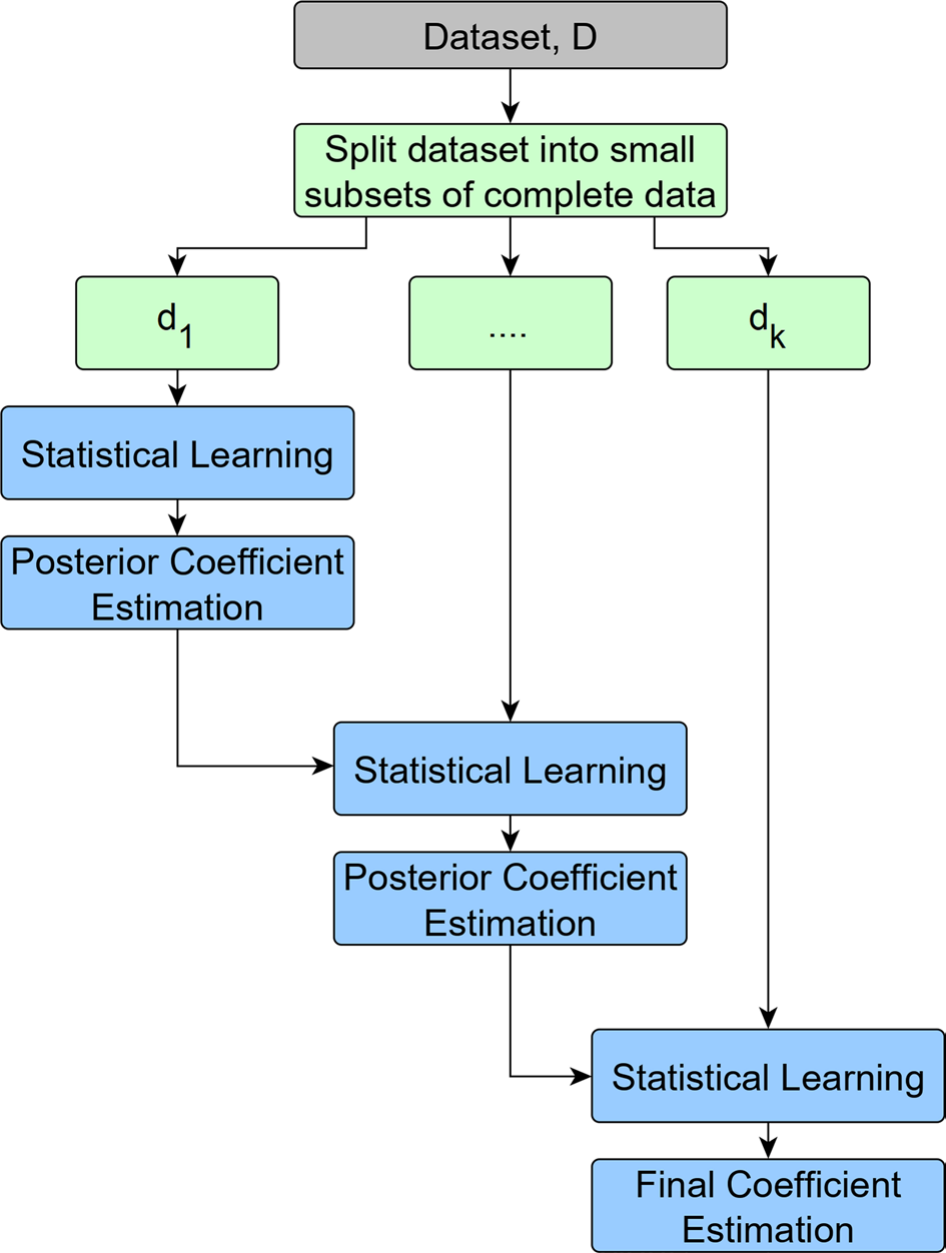
Graphical representation of the Model Updating concept

PMM based imputation is a popular and robust approach for handling MCAR and MAR types of missing data, but it has certain limitations. The DMU approach (Algorithm 1) proposes a different perspective of handling the missing data. While any imputation approach focuses on replacing the missing value with a predicted value to complete the information, DMU approach focuses on building the model on incomplete information rather than on imputed information. The basic framework is to divide the dataset into smaller datasets containing a smaller number of predictors but complete information, and sequentially build the model for each dataset followed by updating the estimates of the predictors after each model, as shown in Fig. 1. It is explained in more details below.

**Table:** 

Algorithm: Dynamic Model Updating	
I	Slice the original dataset *D* with *p* features to create set *d* of *k* datasets, such that: $$d_{k} has no missing values,$$ $$d_{k} \subset D,$$ $$\bigcap {\left( {d_{l} ,d_{m} } \right)} = \emptyset | d_{l} ,d_{m} \in d\; and\; l \ne m$$
II	Sequentially perform Bayesian Regression on each dataset *k* to get posterior estimate, {$\hat{\beta }_{j} {|}j = \left\{ {1, \ldots , p} \right\}$. The posterior estimate of $\hat{\beta }_{j}$ after k-1th dataset is used as the prior estimate for kth dataset

### Dataset fragmentation

A dataset, D with *p* predictor space and *n* total number of samples containing complete outcome data and incomplete predictor data is fragmented into *k* smaller datasets.

6 $$\begin{array}{*{20}c} {D = \left| {\begin{array}{*{20}c} {\begin{array}{*{20}c} {a_{11} } & M \\ \ldots & \ldots \\ {a_{i1} } & \ldots \\ \end{array} } & {\begin{array}{*{20}c} {a_{1j} } & \ldots & {a_{1p} } \\ \ldots & \ldots & \ldots \\ {a_{ij} } & \ldots & M \\ \end{array} } \\ {\begin{array}{*{20}c} M & \ldots \\ \ldots & \ldots \\ {a_{n1} } & \ldots \\ \end{array} } & {\begin{array}{*{20}c} \ldots & M & \ldots \\ \ldots & \ldots & \ldots \\ M & \ldots & {a_{np} } \\ \end{array} } \\ \end{array} } \right| } \\ \end{array}$$

where a_ij_ (s.t. i Є [1, n] and j Є [1, p]) represents the element in dataset D. M represents the element with the missing value. Each of the *k* smaller datasets of D has no missing value but may have reduced predictor space and sample size.

7 $$\begin{array}{*{20}c} {d = \bigcup\limits_{i = 1}^{k} {d_{l} } } \\ \end{array}$$

8 $$\begin{array}{*{20}c} {\bigcap {\left( {d_{l} ,d_{m} } \right)} = \emptyset | d_{l} ,d_{m} \in d\; and\; l \ne m} \\ \end{array}$$

9 $$\begin{array}{*{20}c} {d_{l} = \left| {\begin{array}{*{20}c} {a_{11} } & \ldots & {a_{1c} } \\ \ldots & {a_{22} } & \ldots \\ {a_{r1} } & \ldots & {a_{rc} } \\ \end{array} } \right| | c \in \left[ {1,q} \right], r \in \left[ {1,s} \right], q \in \left[ {1,p} \right], s \in \left[ {1,n} \right]} \\ \end{array}$$

where *k* is the number of subsets in which dataset D is divided and *d* is the set containing k smaller datasets. The *d* set is created such that any of its two elements are mutually exclusive to each other. Any dataset, d_l_ will have maximum *p* predictors and *n* samples. a_rc_ is an element in the dataset d_l_.

#### Hierarchical clustering

Different approaches can segment dataset D into smaller datasets. Literature provides different clustering approaches which can be broadly classified into four types, namely centroid-based, density-based, distribution-based or model-based and connectivity-based [25]. Centroid-based clustering focuses on partitioning samples into clusters with the nearest mean or median [26]. They provide local optima rather than global optima [27]. K-mean clustering is an example of the centroid-based clustering [27]. Density-based clustering focuses on partitioning the samples into clusters with a higher density than the remainder of the samples [28]. Hence many samples may not be assigned any cluster. DBSCAN is an example of the density-based clustering [28].

Distribution-based clustering focuses on partitioning the samples into clusters with similar statistical distribution [29]. They suffer from convergence to local optima and overfitting [30]. Gaussian mixture models is an example of distribution-based clustering [29]. Connectivity-based clustering or hierarchical clustering partitions the samples based on the distance of a sample with other samples. The similar samples have lower distance among them as compared to dissimilar samples. It does not provide a single set of clusters rather a hierarchy of clusters based on the threshold distance value used to partition the data [31]. It is a computationally intensive approach [32]. The current study uses hierarchical clustering to partition dataset D. Hierarchical clustering does not have the issue of local optimum, avoids rejection of sparse samples and does not require the prior knowledge of statistical distribution model for samples.

#### Subgroup construction

**Fig. 2:**
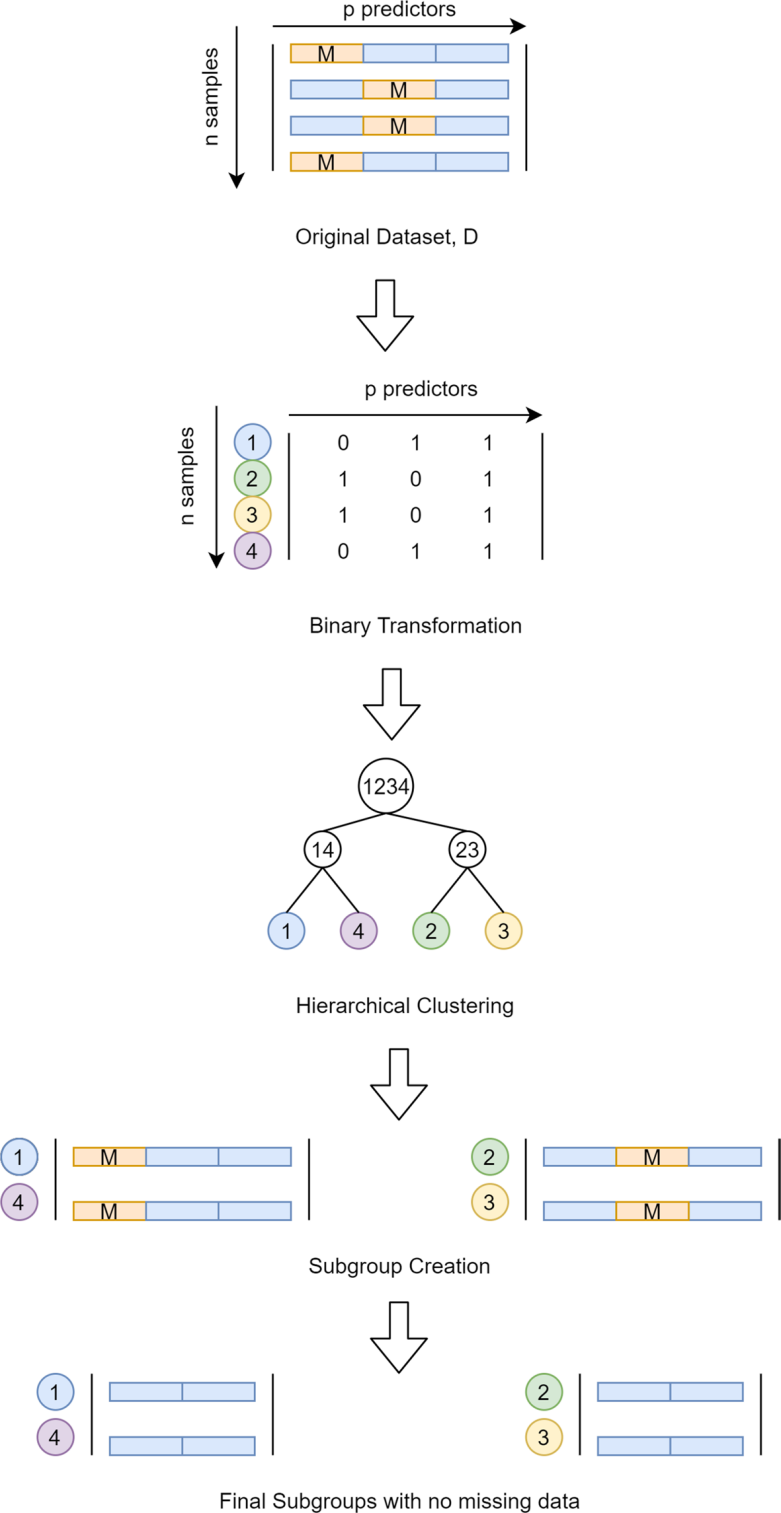
Graphical representation of Subgroup Construction. *M* Mi

The predictor space of dataset D is split into *k* subgroups using hierarchical clustering technique (Fig. 2). The clustering technique needs to classify the samples in D based on the similarity (or, dissimilarity) in the missingness pattern. The dataset D contains mixtures of missing values and non-missing values. The magnitude of non-missing values can influence the clustering computation since hierarchical clustering techniques rely upon the distance between the samples. The magnitude effect of non-missing values is eliminated by transforming the predictor space of dataset D into binary data where a value zero is assigned to a missing value and one is assigned to non-missing values as shown below:

10 $$\begin{array}{*{20}c} {D_{ij} = \left| {\begin{array}{*{20}c} {\begin{array}{*{20}c} {a_{11} } & M \\ \ldots & \ldots \\ {a_{i1} } & \ldots \\ \end{array} } & {\begin{array}{*{20}c} {a_{1j} } & \ldots & {a_{1p} } \\ \ldots & \ldots & \ldots \\ {a_{ij} } & \ldots & M \\ \end{array} } \\ {\begin{array}{*{20}c} M & \ldots \\ \ldots & \ldots \\ {a_{n1} } & \ldots \\ \end{array} } & {\begin{array}{*{20}c} \ldots & M & \ldots \\ \ldots & \ldots & \ldots \\ M & \ldots & {a_{np} } \\ \end{array} } \\ \end{array} } \right|| i \in \left[ {1,n} \right], j \in \left[ {1,p} \right]} \\ \end{array}$$

11 $$\begin{array}{*{20}c} {Bin.D_{ij} = \left| {\begin{array}{*{20}c} {\begin{array}{*{20}c} 1 & 0 \\ \ldots & \ldots \\ 1 & \ldots \\ \end{array} } & {\begin{array}{*{20}c} 1 & \ldots & 1 \\ \ldots & \ldots & \ldots \\ 1 & \ldots & 0 \\ \end{array} } \\ {\begin{array}{*{20}c} 0 & \ldots \\ \ldots & \ldots \\ 1 & \ldots \\ \end{array} } & {\begin{array}{*{20}c} \ldots & 0 & \ldots \\ \ldots & \ldots & \ldots \\ 0 & \ldots & 1 \\ \end{array} } \\ \end{array} } \right| } \\ \end{array}$$

where D_ij_ represents the original dataset with n samples and p predictors, M represents the missing values and Bin.D_ij_ represents the binary transformation of D_ij_ matrix. Hierarchical clustering of Bin.D_ij_ is performed. The *n* rows are used as samples which are to be clustered with *p*-dimensional data.

The number of clusters selected from the hierarchical clustering represents the total number of subgroups, *k* in which dataset D is divided. *k* is the hyperparameter which determines the number of subgroups used in model building and is user-defined.

### Model building

The model building step relies upon the Bayesian paradigm and the assumption that predictors are independent of each other. The Bayesian paradigm focuses on finding the distribution of the parameter estimate of a predictor [33]. The Bayesian paradigm takes the prior belief about the distribution of parameter estimate. This prior belief is updated to give the posterior distribution of parameter estimate of a predictor with likelihood estimate using the data. The posterior distribution of the parameter estimate of a predictor from the previous model can be used as a prior belief for the next model. If the consecutive model contains the same predictor, the prior distribution will then be updated; else it will not. Bayesian regression is used to create a model for each of the dataset, *d*_*l*_. Dynamically, the posterior probability of one model is used as the prior probability for the next model. Only, for the first model, the prior probability for each predictor need to be pre-specified.

### Hyperparameter selection

The hyperparameter *k*, i.e., the total number of subgroups, in the model is user-defined. However, it may not always be possible to know the optimum *k*. In such a scenario, the DMU method could be run for all the possible values of *k*, i.e. from 1 to n, which will generate *n* different models. The model with the best performance is selected as the final model. Researchers can define the performance metric used for selecting the model and, consequently, *k*. In the current study, the performance metric used to evaluate different models is the root mean square error of the model on the test dataset or unknown dataset.

In large datasets, the hyperparameter selection can become computationally intensive. Accordingly, it is desirable to incorporate an optimization algorithm to increase speed and reduce computation intensiveness. Various types of optimization algorithms exist in the literature [34, 35]. The current study chooses Genetic Algorithm (GA), which is a metaheuristic algorithm that does not perform differentials. The algorithm is inspired by the natural evolution process which occurs in living organisms. In summary, GA starts with an initial *population* (or, samples) from the search space and determines their *fitness* (or, performance). Some samples are selected based on their *fitness* value as the *parent population,* which influence the *offspring population* (or new samples). The algorithm relies upon the *crossover* (recombining the parameter values of the selected pair of *parent population*) for convergence and *mutation* (random change in the parameter value of the selected pair of *parent population)* for divergence in the *offspring population*. This process undergoes iteration until the desirable or best performance is achieved. One of its limitations is that it may get stuck in local optimum, but it can provide a good solution for a diversity of problems [36]. In the current study, GA *population* is the value of *k* and *fitness* function is the root mean square error obtained by the Bayesian regression for test dataset.

## Supplementary Information

**Additional file 1.** Table S1: MSE performance of different regression methods in simulated datasets.

**Additional file 2.** Table S2: MSE performance of different regression methods in real datasets.

## Data Availability

All the datasets and code are in the GitHub link: https://github.com/rahijaingithub/DMU.

## Abbreviations

CCA: Complete case analysis
DMU: Dynamic model updating
GA: Genetic Algorithm
K-NN: K-Nearest Neighbors
MCAR: Missing completely at random
MCMC: Markov Chain Monte Carlo
MSE: Mean square error
NCR: No complete row
PMM: Predictive mean matching
SCR: Some complete rows
SLR: Simple linear regression
SLRM: Simple linear regression combined with imputation
