# Supplementary material for: Dynamic model updating (DMU) approach for statistical learning model building with missing data
Source: BMC Bioinformatics. 2021 Apr 29;22:221. doi: 10.1186/s12859-021-04138-z (PMC8086098; doi:10.1186/s12859-021-04138-z)
Supplement: Supplementary file 1 — Additional file 1. Table S1: MSE performance of different regression methods in simulated datasets. [file 12859_2021_4138_MOESM1_ESM.docx]

Additional file 1: Table S1: MSE performance of different regression methods in simulated datasets.

| **Settings** | **p** | **Average MSE (Method) (S=30)** | | | | |
| --- | --- | --- | --- | --- | --- | --- |
|  |  | **SLR^*^(95% CI)** | **KNN^*^(95% CI)** | **SLRM^*^(95% CI)** | **RF^*^(95% CI)** | **DMU^*^(95% CI)** |
| **SCR^*^** | 20 | 0.11(0.11-0.12) | 0.11(0.1-0.11) | 0.23(0.21-0.24) | 0.13(0.12-0.15) | **0.11(0.10-0.12)** |
|  | 25 | 0.13(0.13-0.14) | 0.11(0.1-0.11) | 0.23(0.22-0.25) | 0.13(0.12-0.14) | **0.12(0.12-0.13)** |
|  | 30 | 0.16(0.15-0.17) | 0.11(0.1-0.11) | 0.22(0.21-0.24) | 0.13(0.12-0.14) | **0.11 (0.11-0.12)** |
| **NCR^*^** | 20 | - | - | 0.23(0.21-0.24) | 0.14(0.12-0.15) | **0.17(0.14-0.21)** |
|  | 25 | - | - | 0.24(0.22-0.25) | 0.13(0.12-0.15) | **0.2(0.17-0.24)** |
|  | 30 | - | - | **0.22(0.21-0.23)** | 0.13(0.12-0.14) | 0.23(0.19-0.26) |
| * SLR: Simple Linear Regression, KNN: k Nearest Neighbors based Imputation, SLRM: Simple Linear Regression combined with imputation, RF: Random Forest-based Imputation, DMU: Dynamic Model Updating, SCR: Some Complete Rows in training data, NCR: No Complete Rows in training data, CI: Confidence Interval | | | | | | |
