# Supplementary material for: Dynamic model updating (DMU) approach for statistical learning model building with missing data
Source: BMC Bioinformatics. 2021 Apr 29;22:221. doi: 10.1186/s12859-021-04138-z (PMC8086098; doi:10.1186/s12859-021-04138-z)
Supplement: Supplementary file 2 — Additional file 2. Table S2: MSE performance of different regression methods in real datasets. [file 12859_2021_4138_MOESM2_ESM.docx]

Additional file 2: Table S2: MSE performance of different regression methods in real datasets.

| **Scenario** | **Dataset** | **MSE (Method)/ MSE (Mean Imputation)** | | | | |
| --- | --- | --- | --- | --- | --- | --- |
|  |  | ***SLR*** | ***kNN*** | ***SLRM*** | ***RF*** | ***DMU*** |
| 1 | I | 2.82 | 8.1 | 2.84 | 18.6 | **1.2** |
| 2 | I | 2.48 | 7.9 | 3.16 | 11.4 | **1.1** |
| 3 | I | 5.29 | 11.80 | 9.13 | 13.80 | **0.87** |
| 4 | I | - | - | 10.4 | 8.72 | **4.36** |
| 5 | I | - | - | 5.36 | 7.47 | **1.14** |
| 6 | I | - | - | 25.40 | 32.70 | **1.00** |
| 7 | II | 4.66 | 5.31 | 4.65 | 5.53 | **4.65** |
| 8 | II | 3.37 | 10.2 | 3.89 | 6.37 | **3.34** |
| 9 | II | 1.16 | 4.15 | 1.48 | 2.7 | **1.15** |
| 10 | II | - | - | 6.02 | 11.7 | **4.77** |
| 11 | II | - | - | 10.6 | 7.07 | **4.01** |
| 12 | II | - | - | 3.73 | 2.73 | **1.95** |
